# Enhanced Pepper Resistance to *Sclerotium rolfsii* Through Root Development and Enzyme Modulation by Hexaconazole and Azoxystrobin

**DOI:** 10.1002/pld3.70136

**Published:** 2026-01-11

**Authors:** Yanlong Jia, Rong Wen, Chuanjing Liang, Xiaolong Lan, Tingting Mao, Dan Xing, Wenjie Lin

**Affiliations:** ^1^ School of Chemistry and Environmental Engineering Hanshan Normal University Chaozhou China; ^2^ Institute of Pepper Guizhou Academy of Agricultural Sciences Guiyang China; ^3^ College of Agriculture Guizhou University Guiyang China; ^4^ Institute of Plant Protection Guizhou Academy of Agricultural Sciences Guiyang China

**Keywords:** azoxystrobin, hexaconazole, pepper, root, *Sclerotium rolfsii*

## Abstract

Southern blight, caused by the soil‐borne fungus *Sclerotium rolfsii* (*S. rolfsii*), poses a significant threat to pepper ( *Capsicum annuum* L.) production, necessitating the development of effective chemical control strategies. This study investigated the physiological responses of pepper plants to *S. rolfsii* infection and evaluated the efficacy of the fungicides hexaconazole and azoxystrobin. The results demonstrated that hexaconazole, applied at 50 μg·mL^−1^, provided outstanding protective activity (97.56%). In contrast, azoxystrobin, at a higher concentration of 100 μg·mL^−1^, exhibited optimal overall control, with 88.62% protective and 49.06% curative activity. Beyond direct pathogen suppression, both fungicides mitigated disease impact by safeguarding host plant growth, promoting root system development, and enhancing defense responses through the induction of key antioxidant enzymes, namely, peroxidase (POD) and catalase (CAT). Consequently, the application of hexaconazole and azoxystrobin significantly reduced disease progression and protected normal plant growth. These findings provide a scientific basis for effective management of southern blight in pepper and elucidate how fungicides with distinct modes of action can enhance plant resistance by modulating the antioxidant system.

**Abbreviations**
ATP: adenosine triphosphate
CAT: catalase
DI: disease index
DMSO: dimethyl sulfoxide
MDA: malondialdehyde
POD: peroxidase
ROS: reactive oxygen species
SPAD: soil plant analysis development

## 1 Introduction

The soil‐borne fungus *Sclerotium rolfsii* (*S. rolfsii*) is a highly destructive pathogen with a broad host range, causing southern blight disease in over 500 plant species and leading to severe crop yield losses (Paparu et al. 2020). Pepper ( *Capsicum annuum* L.) is among the economically important crops susceptible to *S. rolfsii* (Qiu et al. 2024; Song et al. 2024). Current management strategies for *S. rolfsii* include the deployment of resistant varieties (Yelin et al. 2022), biological control (Jacob et al. 2018), and chemical control (Han et al. 2023). However, the availability of commercially acceptable resistant pepper varieties is limited, and the development of such varieties through breeding is a time‐consuming process. Although environmentally friendly, biological control methods often lack validation through large‐scale, long‐term field trials (Xin et al. 2020). Consequently, chemical control, primarily through the application of fungicides, remains the most widely adopted strategy for managing *S. rolfsii* (F. Wang et al. 2023). A significant challenge associated with heavy reliance on chemical control is the risk of pathogens developing resistance, particularly to fungicides with a single‐site mode of action (Rancāne et al. 2023). Therefore, the screening and development of efficient fungicides with diverse mechanisms of action are crucial for sustainable *S. rolfsii* management and are essential for formulating cost‐effective, potent fungicidal compositions.

Ergosterol and mitochondria play pivotal roles in fungal biology. Ergosterol, an essential sterol component of the cell membrane, is critical for maintaining its fluidity, integrity, and permeability (Tlapale‐Lara et al. 2024). Mitochondria, as the primary organelles for cellular respiration, are fundamental to energy metabolism, a vital process sustaining fungal growth and development (Jin et al. 2021). The triazole fungicide hexaconazole offers broad‐spectrum protective and therapeutic effects by disrupting ergosterol biosynthesis in fungi, preventing fungal cell membrane formation, and leading to fungal death (Abdi et al. 2021), and exerts a potent inhibitory effect on fungal toxins (Kumar et al. 2016; C. Li et al. 2022). Hexaconazole effectively controls southern blight disease in pepper through in vitro fungal inhibitory activity (Das et al. 2014). Azoxystrobin, a synthetic methoxyacrylate fungicide (QoI), exerts its fungicidal effect by competitively binding to the Qo site of the pathogenic fungal mitochondrial cytochrome *bc*_1_ complex via its methoxyacrylate pharmacophore. This blockade of mitochondrial electron transport triggers lethal energy failure and oxidative stress in fungal cells (Fisher et al. 2020). However, little research exists on the differential control effects of hexaconazole and azoxystrobin against *S. rolfsii*. Therefore, exploring the control effects is vital for *S. rolfsii* management and lays the foundation for the subsequent fungicide compounding.

Plant root systems can alter their morphology to adapt to environmental stresses (Williams and de Vries 2020) and regulate the antioxidant system to scavenge reactive oxygen species (ROS), a toxic byproduct of adversity metabolism (Choudhury et al. 2017; J. Zhang et al. 2021). Fungicides not only inhibit fungi and protect stable root growth (Song et al. 2024) but also induce the expression of plant antioxidant enzymes (Nong et al. 2021), thereby enhancing plant disease resistance. Studies have shown that hexaconazole induces morphological and physiological changes such as reduced shoot growth, stimulated root growth (Navarro et al. 2009), and induction of antioxidant defenses in plants to ameliorate stress (Akbari et al. 2011). Azoxystrobin induces enhanced antioxidant enzyme activity and protects plants from harmful ROS (Liang et al. 2018), inducing delayed senescence (Y. Zhang et al. 2010). Both fungicides have been shown to modulate plant antioxidant defenses and morphology, suggesting a common potential to enhance plant resilience. Therefore, exploring the changes in the root system of pepper under *S. rolfsii* infection by application of hexaconazole and azoxystrobin could provide a theoretical basis for the control of southern blight disease.

This study evaluated the efficacy of the triazole fungicide hexaconazole and the methoxyacrylate fungicide azoxystrobin against *S. rolfsii* in pepper under controlled pot conditions. The primary objectives were to (i) compare the differential control effects of these two fungicides with distinct modes of action on *S. rolfsii* and (ii) identify their optimal application concentrations. This work aims to provide a scientific basis for the chemical prevention and control of pepper southern blight. Furthermore, the findings may offer valuable insights for the future development of compound fungicides based on synergistic principles.

## 2 Materials and Methods

### 2.1 Strain, Plant, and Fungicide

The fungal pathogen *S. rolfsii* was obtained from the fungal culture collection at the Capsicum Research Institute of Guizhou Academy of Agricultural Sciences, China. Stock cultures were maintained on potato dextrose agar (PDA) plates and incubated under controlled conditions (25°C ± 1°C) at the Fine R&D Centre of Guizhou University. For long‐term preservation, cultures were stored at 4°C. The experimental plant material consisted of *C. annuum* “Qianjiao 8” chili pepper (cultivar), sourced from the Institute of Pepper, Guizhou Academy of Agricultural Sciences, Guizhou Province. All seedlings were cultivated in a greenhouse environment under strictly standardized growth conditions to ensure uniformity. The plants were cultivated in a controlled environment with the temperature maintained at 25°C, a 16/8 h (light/dark) photoperiod, and a light intensity ranging from 700 to 1100 lx. Experiments were performed when the pepper seedlings reached the sixth to eighth true‐leaf stage. Only seedlings exhibiting identical growth potential were selected for the trials. Two commercially available fungicides from different chemical classes were used in this study. Hexaconazole, a triazole‐class fungicide, was obtained from Shanghai Yuanye Biotechnology Co. Ltd., with a certified active ingredient purity of ≥ 95%. Azoxystrobin, a strobilurin (QoI) class fungicide, was purchased from Shanghai Macklin Biochemical Technology Co. Ltd., with a certified active ingredient purity of ≥ 98%.

### 2.2 Pot Test of Hexaconazole and Azoxystrobin Against *S. rolfsii*

#### 2.2.1 Disease Index (DI) Assessment

To rigorously quantify the impact of Southern blight caused by *S. rolfsii* on chili pepper plants, a standardized disease assessment protocol adapted from established methods was employed (Song et al. 2024). Disease severity was quantified by calculating a DI based on a 0–10 rating scale (Table 1).

**TABLE 1:** Standardized scale for quantifying *S. rolfsii* disease severity on chili pepper.

Rating level	Symptom description	Severity percentage
0	Asymptomatic. No visible stem browning, foliage is healthy.	0%
1	Minor stress. No visible stem browning; however, slight, localized foliar chlorosis or leaf yellowing is present.	10%
3	Initial stem lesion. Slight basal stem browning and shriveling are observed. Foliar symptoms include general chlorosis or initial yellowing.	30%
5	Moderate lesion progression. Basal stem lesion extends up to 1 cm. Foliage exhibits notable shriveling (wilting) or initial leaf abscission (drop).	50%
7	Significant lesion progression. Basal stem lesion ranges from 1 to 2 cm in length. Severe leaf shriveling or widespread leaf drop is evident.	70%
9	Severe lesion progression. Basal stem lesion ranges from 2 to 3 cm in length. Plant exhibits near‐total wilting and imminent death.	90%
10	Plant mortality. Basal stem lesion is significant (greater than 3 cm); the entire plant is wilted and deceased.	100%

The DI—a quantitative measure of disease severity across the treatment population—was calculated using the following formula:

$$\mathrm{DI}\;\left(\%\right)=\frac{\sum \left(\text{number of diseased plants}\;\mathrm{at}\;\text{each level}\times \text{number of corresponding levels}\right)}{\text{total number of plants surveyed}\times \text{highest level value}}\times 100\%,$$

where ∑ represents the summation across all observed rating levels (0, 1, 3, 5, 7, 9, 10).

#### 2.2.2 In Vivo Curative and Protective Activity

The mycelial culture and inoculation procedures were adapted from a previously established method (Song et al. 2024). Five *S. rolfsii* discs were added to PDB medium and placed on a shaker (25°C, 180 rpm) in the dark for 4 days. Freshly harvested mycelia were subjected to three sequential rinses with sterile water and subsequently isolated via filtration. Then, 0.5 g of fresh mycelium was placed in the soil at a 1‐cm position of pepper roots. The roots of pepper were treated once with fungicide for protection/curative activity by root irrigation (5 mL a plant) at 24 h before and 24 h after inoculation, respectively. The control group (CK) received a treatment of 1% dimethyl sulfoxide (DMSO), sterile distilled water, and 0.1% Tween 20. Both hexaconazole and azoxystrobin were set at concentrations of CK (0 μg·mL^−1^), 50 μg·mL^−1^, and 100 μg·mL^−1^. The solvent control (1% DMSO and 0.1% Tween 20) was confirmed to have no significant effect on fungal growth or plant development in preliminary experiments. The experiment was arranged in a completely randomized block design under greenhouse conditions. Each treatment consisted of five biological replicates, with each replicate comprising one uniformly grown pepper plant. Throughout the experimental period, plants were regularly observed and visually assessed for any phytotoxic symptoms, such as chlorosis, malformation, or necrosis on leaves, stems, or other tissues. These observations were systematically recorded. DI was assessed on the fourth day of *S. rolfsii* infection, and control effect was calculated. Control effect (%) = [(DI of the blank group—DI of the treatment group)/DI of the blank group] × 100%.

### 2.3 Effect of Hexaconazole and Azoxystrobin on Plant Height and Chlorophyll (SPAD) of *S. rolfsii*‐Infected Pepper Plants

Within the treatments outlined in Section 2.2.2, plant height was measured before and on Day 4 posttreatment using the straightedge method, with the straightedge positioned vertically against the stem base and plant height read directly from the scale. The relative chlorophyll content was quantified using the SPAD‐502 Plus portable chlorophyll meter (Konica Minolta, Japan). Measurements were uniformly taken on the third or fourth layer of leaves, counted from the top to the bottom, between 08:00 and 11:00 a.m. Each leaf was measured at three positions (base, middle, and tip), with each position measured three times, and the overall average taken as the chlorophyll (SPAD) value for the leaf.

### 2.4 Effect of Hexaconazole and Azoxystrobin on Root Morphology of *S. rolfsii*‐Infected Pepper Plants

To evaluate the impact of treatments, root system architecture (RSA) was characterized 4 days after treatment application. Root samples were collected, digitized using a GXY root scanner, and the resulting digital images were analyzed with WhizoPheno software. This approach yielded quantitative data on key growth metrics: root length, surface area, volume, and the count of root apices.

### 2.5 Effect of Hexaconazole and Azoxystrobin on Root Enzyme Activities of *S. rolfsii*‐Infected Pepper Plants

Posttreatment in Section 2.2.2, root samples were collected at Day 4 and immediately frozen in liquid nitrogen. The samples were ground to a fine powder, and total protein was extracted. The protein concentration was determined according to the Bradford method using a kit (Soleberg Biotechnology, Beijing, China) following the manufacturer's instructions. Peroxidase (POD) activity (product number: bc0095, micromethod, 100T/96S) and catalase (CAT) activity (product number: bc0205, micromethod, 100T/96S) were then assessed using a kit from Soleberg Biotechnology, Beijing, China, according to the manufacturer's instructions. Enzyme activities were expressed as units per gram of protein (U·g^−1^ protein). Malondialdehyde (MDA) content was determined and expressed as nanomoles per gram of fresh weight (nmol·g^−1^ FW). Absorbance was measured using a microplate detector (Maltiskan, China).

### 2.6 Statistical Analysis

The experiment was set up with five biological replicates. All data represent means of five independent replicates, and data analysis was conducted using SPSS 26.0 statistical software through ANOVA, with *p* ≤ 0.05 considered significant.

## 3 Results

### 3.1 DI of Hexaconazole and Azoxystrobin on *S. rolfsii*‐Infected Pepper Plants

**FIGURE 1:**
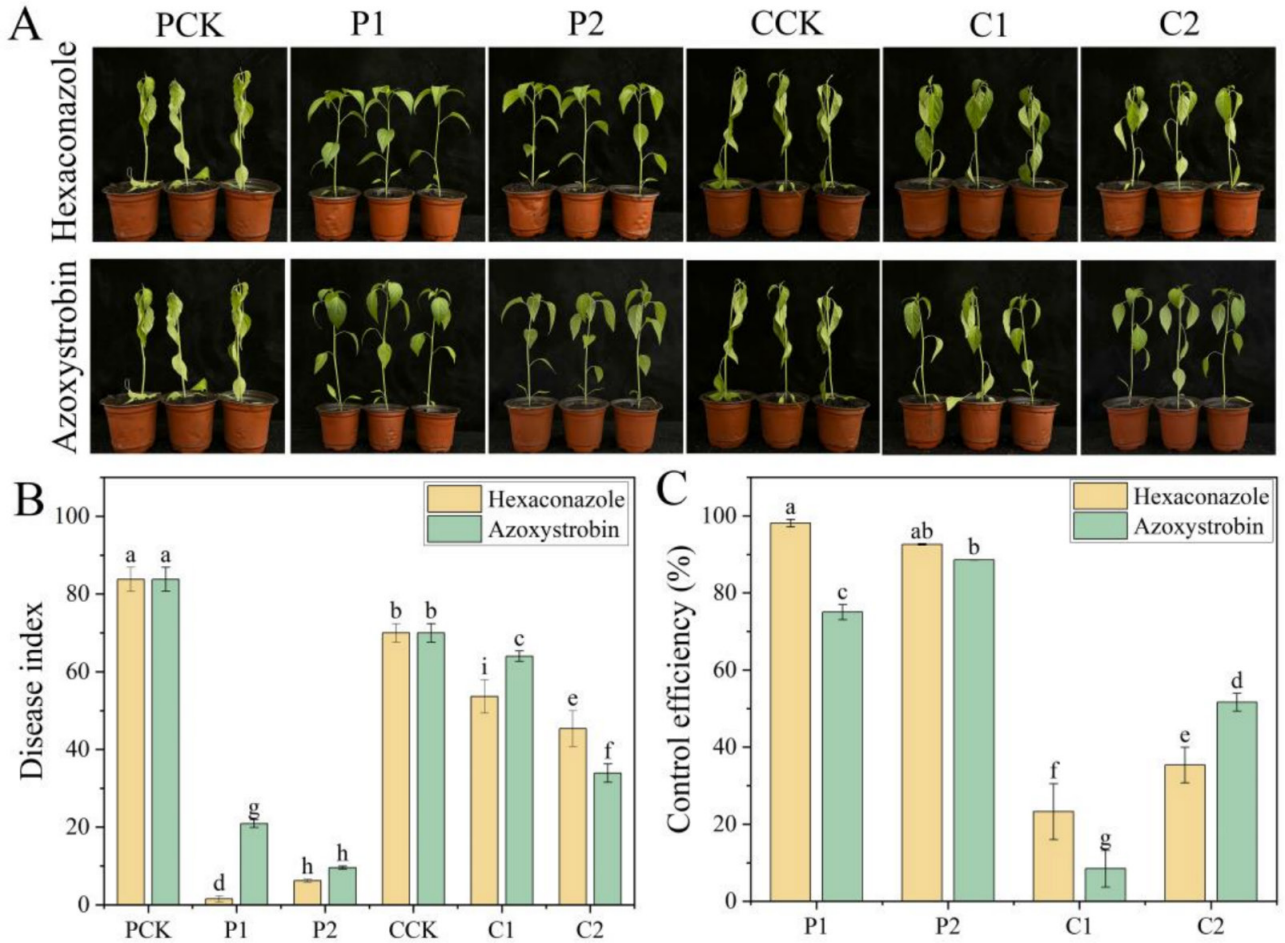
In vivo protective and curative activities of hexaconazole and azoxystrobin against *S. rolfsii*. Differences in growth (A), disease index (B), and control effect (C) among treatments. PCK represents the control group for protective activity, P1 represents protective activity with fungicide at 50 μg·mL^−1^, P2 represents protective activity with fungicide at 100 μg·mL^−1^, CCK represents the control group for curative activity, C1 represents curative activity with fungicide at 50 μg·mL^−1^, and C2 represents curative activity with fungicide at 100 μg·mL^−1^. Data are means ± SE (*n* = 5). Different letters indicate significant differences at *p* < 0.05. The same is applied to other figures below.

At 4 days post *S. rolfsii* infection, the protective/curative activity indices in the CK group reached 82.0 and 70.7, respectively, with plants exhibiting chlorosis due to reduced chlorophyll, followed by shriveling and subsequent leaf abscission (Figure 1A,B). Hexaconazole demonstrated a protective activity of 97.6% and 92.7% at concentrations of 50 and 100 μg·mL^−1^, respectively. Under the same conditions, the protective activity of azoxystrobin was 74.8% at 50 μg·mL^−1^ and 88.6% at 100 μg·mL^−1^. Regarding curative activity, hexaconazole achieved 31.1% and 32.1% efficacy at 50 and 100 μg·mL^−1^, respectively, whereas azoxystrobin showed 11.3% and 49.1% efficacy at the corresponding concentrations (Figure 1C). This result showed that hexaconazole and azoxystrobin were superior to the curative activity at the same concentration. Hexaconazole was superior to azoxystrobin for protective activity; for curative activity, hexaconazole was superior to azoxystrobin at low concentration (50 μg·mL^−1^), and azoxystrobin was superior to hexaconazole at high concentration (100 μg·mL^−1^).

### 3.2 Effect of Hexaconazole and Azoxystrobin on Plant Height and Chlorophyll (SPAD) of *S. rolfsii*‐Infected Pepper Plants

**FIGURE 2:**
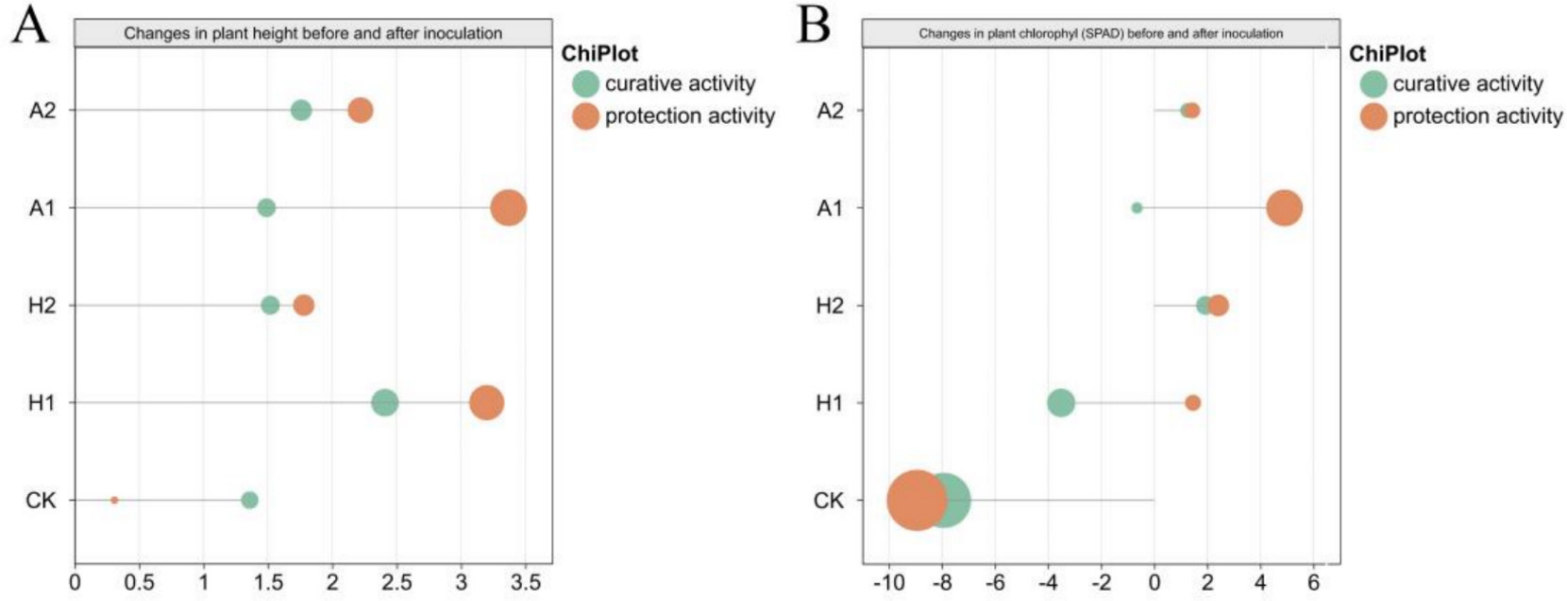
Effects of hexaconazole (H) and azoxystrobin (A) on plant height (A) and chlorophyll content (B) in pepper plants infected with *S. rolfsii*. H: hexaconazole; A: azoxystrobin; CK: control group; 1: 50 μg·mL^−1^; 2: 100 μg·mL^−1^.

To evaluate the effect of hexaconazole and azoxystrobin on *S. rolfsii*‐infected pepper plants, plant height and chlorophyll indexes were determined before and after inoculation, with growth changes during the period are shown in Figure 2. The results showed that hexaconazole and azoxystrobin sustained plant growth and maintained chlorophyll stability to varying extents as compared with the control.

### 3.3 Effect of Hexaconazole and Azoxystrobin on Root Morphology of *S. rolfsii*‐Infected Pepper Plants

**FIGURE 3:**
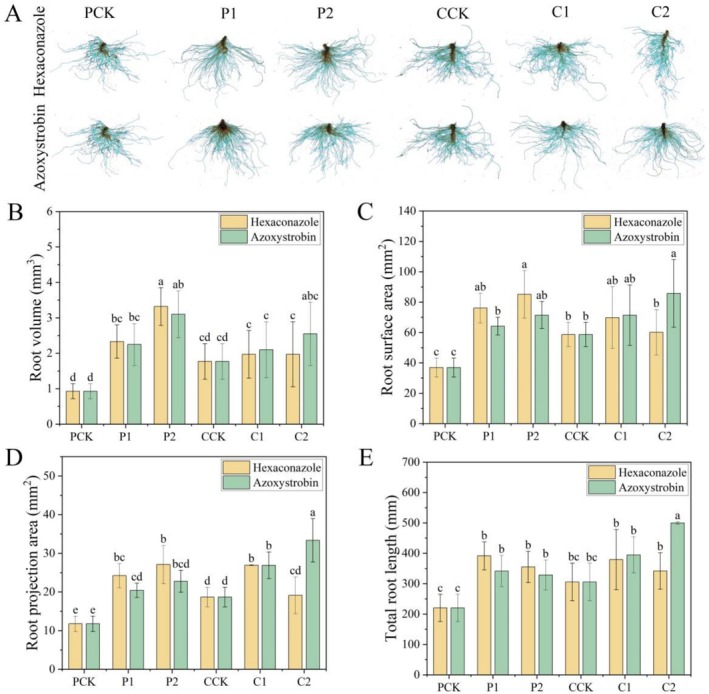
Quantitative comparison of root system architecture metrics across treatment groups (A) and detailed analysis of root volume (B), root surface area (C), root projection area (D), and total root length (E) in pepper plants under *S. rolfsii*. Treatments are consistent with those described in Figure 1.

The impact of hexaconazole versus azoxystrobin application on RSA following *S. rolfsii* infection of pepper was examined as shown in Figure 3. Compared with CK, hexaconazole application induced the highest increase in total root length (1.78‐fold), root volume (3.57‐fold), root projected area (2.31‐fold), and root surface area (2.31‐fold), while azoxystrobin induced the highest increase in total root length (1.55‐fold), root volume (3.33‐fold), root projected area (1.94‐fold), and root surface area (1.94‐fold). The results demonstrate that both fungicides ameliorated the root growth inhibition caused by *S. rolfsii* stress, leading to significant improvements in total root length, root volume, and root projected and surface areas. Furthermore, a positive correlation was observed between the concentration required for effective disease control and the magnitude of the growth‐promoting effect on the root system.

### 3.4 Effect of Hexaconazole and Azoxystrobin on Enzyme Activities of *S. rolfsii*‐Infected Pepper Roots

**FIGURE 4:**
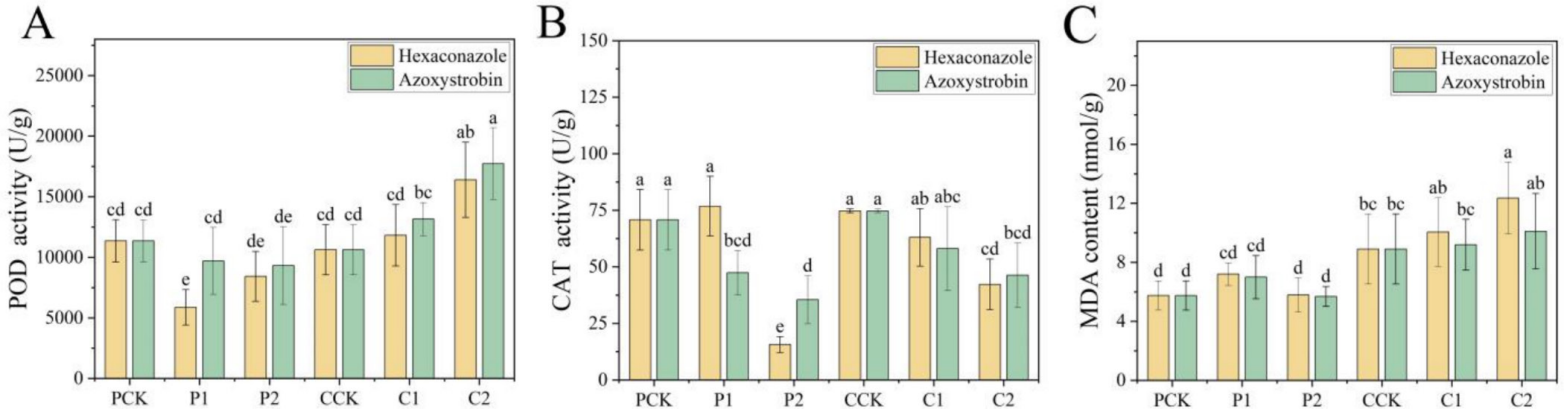
Effects of hexaconazole and azoxystrobin on enzyme activities in roots of *S. rolfsii*‐infected pepper plants: peroxidase (POD) activity (A), catalase (CAT) activity (B), and malondialdehyde (MDA) content (C). Treatments are consistent with those described in Figure 1.

The response of root enzyme activities of *S. rolfsii*‐infected pepper under different treatments was evaluated on the fourth day of infestation. The results showed that POD activity increased with the rise of fungicide concentration (Figure 4A), with mean values ranging from 6000 ± 1660 to 17,500 ± 3000 U·g^−1^ protein (*p* < 0.05 between specific groups, e.g., CK vs. hexaconazole 100 μg·mL^−1^). CAT activity decreased with the rise of fungicide concentration (Figure 4B), with mean values ranging from 15.3 ± 3.57 to 78.6 ± 14.3 U·g^−1^ protein (*p* < 0.05 between specific groups, e.g., CK vs. hexaconazole 100 μg·mL^−1^). MDA content increased with the rise of fungicide concentration (Figure 4C), reaching a peak of 12.1 ± 2.60 nmol·g^−1^ FW in the hexaconazole 100 μg·mL^−1^ treatment group. It is evident that under *S. rolfsii* stress, fungicide application can enhance plant defense mechanisms against pathogen infection by inducing root antioxidant enzymes, where hexaconazole focuses on the regulation of CAT and azoxystrobin focuses on the regulation of POD, and the mechanisms of regulation‐induced plant resistance are not consistent between the two.

### 3.5 Phytotoxicity Assessment

Visual observation for phytotoxicity was conducted regularly throughout the study period. No visible phytotoxic symptoms—including chlorosis, leaf malformation, or necrosis—were observed on the aboveground parts of pepper plants across all treatment levels of hexaconazole and azoxystrobin.

## 4 Discussion

### 4.1 Effect of Hexaconazole and Azoxystrobin on the Control of *S. rolfsii*‐Infected Pepper Plants

The soil‐borne fungus *S. rolfsii*, with its long‐lived sclerotia and adaptable hyphae, poses a significant challenge to crop protection, making chemical control a primary management strategy. Triazole fungicides, such as hexaconazole, and methoxyacrylate fungicides (strobilurins), such as azoxystrobin, are broad‐spectrum agents effective against various fungal diseases (Y. Wang et al. 2022; X. Zhang, Ye, et al. 2024; Zobir et al. 2021). Both have demonstrated inhibitory activity against *S. rolfsii* in crops like peanut and stevia (Aggarwal et al. 2010; Koehler and Shew 2017; Mehan et al. 1994; Rideout et al. 2002; Sanabria‐Velazquez et al. 2023). In this study, both fungicides significantly reduced the severity of southern blight in pepper and inhibited the pathogen, consistent with reports of their efficacy against diseases in pomegranate, rice, and other hosts (Alquraini 2023; Kiiker et al. 2021; Liu et al. 2024). Hexaconazole (50 μg·mL^−1^) showed superior protective (97.6%) and curative activity, whereas azoxystrobin exhibited concentration‐dependent effects, with higher efficacy at 100 μg·mL^−1^.

The distinct efficacy profiles—with hexaconazole providing strong early protection and azoxystrobin offering concentration‐dependent curative potential—offer a rationale for future investigation into combination strategies. However, the assessment at a single early time point (4 days post inoculation) offers a snapshot of initial efficacy but limits insight into effect longevity. Future work should involve multiple time points to elucidate long‐term efficacy and durability of control. Furthermore, the occurrence of isolates with reduced sensitivity at this early stage highlights a potential resistance risk. As this study evaluated only two concentrations, establishing complete dose–response relationships and precise *EC*_50_ values for local populations should be a priority for future resistance monitoring. The inclusion of a standard recommended fungicide as a positive control in subsequent experiments would also provide a valuable efficacy benchmark.

### 4.2 Effect of Hexaconazole and Azoxystrobin on the Growth of *S. rolfsii*‐Infected Pepper Plants

Fungal diseases disrupt normal plant growth (Cai et al. 2022; Xue et al. 2018), while effective fungicides can mitigate damage and positively influence host physiology (Ferreira et al. 2014). Beyond pathogen suppression, they can enhance chlorophyll content (J. Li et al. 2021), improve host resistance, and modulate photosynthetic efficiency and metabolic profiles (C. Zhang et al. 2022), ultimately supporting yield and quality (Fanning et al. 2022). Our results align with these findings: Application of hexaconazole or azoxystrobin under *S. rolfsii* stress helped maintain pepper plant growth, chlorophyll stability, and root system development—increasing total root length, root volume, projected area, and surface area. Additionally, by inducing POD and CAT activities, both fungicides likely enhanced the plant's antioxidant capacity, alleviating oxidative damage caused by the pathogen (L. Zhang, Fang, et al. 2024). The differential induction of POD and CAT suggests they modulate defense pathways differently, a premise requiring molecular validation.

The distinct physiological impacts induced by hexaconazole and azoxystrobin, likely stemming from their differential induction of antioxidant enzymes and modulation of defense pathways, provide a compelling rationale for future investigations. First, the concentration range tested identified effective doses but a broader gradient would refine application guidelines and clarify full dose–response dynamics. Second, more sensitive phytotoxicity assessments (e.g., chlorophyll fluorescence, photosynthetic parameters, and biochemical markers) are needed to comprehensively evaluate potential sublethal effects. Finally, the differential physiological impacts induced by hexaconazole and azoxystrobin, stemming from their distinct modes of action, present a compelling rationale for future studies on their combined use. Such research should rigorously assess interactions using established models (e.g., Colby's method). Validation under diverse field conditions, including residue and long‐term environmental impact studies, remains an essential step toward translating these findings into sustainable disease management strategies for pepper production.

## 5 Conclusions

This study validated the efficacy of the triazole fungicide hexaconazole and the methoxyacrylate fungicide azoxystrobin against Southern blight caused by *S. rolfsii* in pepper. Both fungicides significantly suppressed disease development and demonstrated a strong inhibitory effect on the pathogen. Hexaconazole exhibited optimal protective activity (97.6%) at 50 μg·mL^−1^, whereas azoxystrobin, applied at 100 μg·mL^−1^, showed higher combined efficacy, with 88.6% protective and 49.1% curative activity. Furthermore, both compounds enhanced normal plant growth, indicating their potential as effective agents for managing *S. rolfsii*. Due to their distinct modes of action, the performance of hexaconazole and azoxystrobin can be inconsistent when applied individually. Consequently, formulating them into a combined product may enhance overall efficacy and reliability. These findings provide a theoretical basis for employing hexaconazole and azoxystrobin in the control of *S. rolfsii* on pepper and offer valuable insights for future research on fungicide combination strategies.

## Author Contributions

**Yanlong Jia:** conceptualization, data curation, writing – original draft, visualization, funding acquisition. **Rong Wen:** methodology, formal analysis, investigation, data curation. **Chuanjing Liang:** validation, project administration. **Xiaolong Lan:** validation, formal analysis. **Tingting Mao:** resources, funding acquisition. **Dan Xing:** conceptualization, writing – original draft, visualization, funding acquisition. **Wenjie Lin:** conceptualization, writing – review and editing, supervision, funding acquisition.

## Funding

This research was funded by the construction project of Guizhou Provincial Pepper Industry Technology System (No. GZSLJCYTX‐2025‐03), the Guizhou Provincial Basic Research Program (Natural Science) (No. ZK[2023]General item 191), the Guizhou Provincial Key Technology R&D Program ([2021]492), the Project of Educational Commission of Guangdong Province of China (No. 2023KSYS007, 2023KTSCX078, 2023KCXTD023, 2021ZDJS040, and 2020ZDZX1032), and the Project of Hanshan Normal University (No. XSDBF2025202).

## Conflicts of Interest

The authors declare no conflicts of interest.

## Data Availability

The datasets generated during and/or analyzed during the current study are available from the corresponding author on reasonable request.
